# GRK5 as a Novel Therapeutic Target for Immune Evasion in Testicular Cancer: Insights from Multi-Omics Analysis and Immunotherapeutic Validation

**DOI:** 10.3390/biomedicines13071775

**Published:** 2025-07-21

**Authors:** Congcong Xu, Qifeng Zhong, Nengfeng Yu, Xuqiang Zhang, Kefan Yang, Hao Liu, Ming Cai, Yichun Zheng

**Affiliations:** 1 The Second Affiliated Hospital, Zhejiang University School of Medicine, Hangzhou 310009, China; 21618215@zju.edu.cn (C.X.); liuhao870102@zju.edu.cn (H.L.); 2 The Fourth Affiliated Hospital, Zhejiang University School of Medicine, Yiwu 322000, China; 22218512@zju.edu.cn (Q.Z.); 22318165@zju.edu.cn (X.Z.); 22218509@zju.edu.cn (K.Y.); 3 The First Affiliated Hospital of Zhejiang Chinese Medical University (Zhejiang Provincial Hospital of Chinese Medicine), Hangzhou 310000, China; 22118019@zju.edu.cn

**Keywords:** testicular cancer, potential therapeutic targets, genomics, immune-related cancer subtypes, predictive diagnostic model

## Abstract

**Background:** Personalized anti-tumor therapy that activates the immune response has demonstrated clinical benefits in various cancers. However, its efficacy against testicular cancer (TC) remains uncertain. This study aims to identify suitable patients for anti-tumor immunotherapy and to uncover potential therapeutic targets in TC for the development of tailored anti-tumor immunotherapy. **Methods:** Consensus clustering analysis was conducted to delineate immune subtypes, while weighted gene co-expression network analysis (WGCNA), least absolute shrinkage and selection operator (LASSO) regression, and support vector machine (SVM) algorithms were employed to evaluate the potential efficacy of anti-tumor immunotherapy. Candidate immunotherapy targets were systematically identified through multi-gene panel analyses and subsequently validated using molecular biology assays. A prioritized target emerging from cellular screening was further evaluated for its capacity to potentiate anti-tumor immunity. The therapeutic efficacy of this candidate was rigorously confirmed through a comprehensive suite of immunological experiments. **Results:** Following systematic screening of five candidate genes (WNT11, FAM181B, GRK5, FSCN1, and ECHS1), GRK5 emerged as a promising therapeutic target for immunotherapy based on its distinct functional and molecular associations with immune evasion mechanisms. Cellular functional assays revealed that GRK5 knockdown significantly attenuated the malignant phenotype of testicular cancer cells, as evidenced by reduced proliferative capacity and invasive potential. Complementary immunological validation established that specific targeting of GRK5 with the selective antagonist GRK5-IN-2 disrupts immune evasion pathways in testicular cancer, as quantified by T-cell-mediated cytotoxicity. **Conclusions:** These findings position GRK5 as a critical modulator of tumor-immune escape, warranting further preclinical exploration of GRK5-IN-2 as a candidate immunotherapeutic agent.

## 1. Introduction

Starting from the success of anti-CTLA-4 antibody therapy in malignant melanoma, targeted immunotherapy has emerged as one of the most effective strategies for anti-tumor therapy, igniting a new wave of research in tumor immunotherapy [1]. The 2018 Nobel Prize in Physiology or Medicine was awarded jointly to James P. Allison and Tasuku Honjo “for their discovery of cancer therapy by inhibition of negative immune regulation”. Despite the widespread clinical application of immune checkpoint regulation therapy, its efficacy is limited in many cases, particularly in solid tumors, where the response rate remains low [2,3]. Another popular immunotherapy, adoptive T-cell therapy, is prone to inducing cytokine release syndrome and demonstrates poor persistence in vivo [4].

Significant advances in sequencing technology and bioinformatics algorithms have now enabled researchers to analyze large datasets to identify potential immunotherapeutic targets specifically expressed in tumor tissues. These targets can actively stimulate the autoimmune system and generate an anti-tumor response [5].

Testicular cancer (TC) primarily affects young men aged 20–40 years [6]. Orchidectomy is the standard treatment for this condition, yet many young men find this surgical approach psychologically unacceptable [6]. Given this reality, we aim to explore the potential of conservative chemotherapy to cure or manage TC. For metastatic testicular cancer (mTC), although approximately half of patients respond to salvage therapy, the remainder experience relapse, with poor outcomes. Adverse prognostic features of metastatic lesions and cisplatin resistance mechanisms constitute the primary causes of mortality in most patients. Furthermore, long-term survivors exhibit significantly elevated risks of second primary malignancies, cardiovascular events, and metabolic syndrome. Platinum-based agents also commonly cause non-lethal complications, including dose-dependent hearing impairment, neurotoxicity, and fertility impairment [7,8]. In recent years, however, significant progress has been made in immunotherapy, particularly with the use of anti-PD-1/PD-L1 inhibitors, across various malignancies [9]. This progress has sparked considerable interest in their potential application for treating TC [10]. Research has demonstrated that testicular tumor cells express PD-L1, thereby providing a theoretical foundation for the use of PD-1/PD-L1 inhibitors in this context [11]. To date, anti-PD-1 drugs such as pembrolizumab have been employed to treat patients with platinum-refractory TC, with initial results indicating some degree of efficacy [12].

In addition to PD-1/PD-L1 inhibitors, researchers are actively investigating other immune checkpoint inhibitors, including CTLA-4 and LAG-3 [13,14,15]. Although the preliminary findings are promising, clinical trials assessing the efficacy of immunotherapy for TC remain scarce. Many of these studies are in the early stages and involve small sample sizes, highlighting the necessity for further large-scale randomized controlled trials to validate their efficacy and safety.

Identifying patient populations that could benefit from immunotherapy is currently a critical area of research. The discovery of reliable biomarkers is essential for identifying patients who are likely to respond to immunotherapy, thereby enhancing the precision and effectiveness of treatment.

In this study, the “ConcensusClusterPlus” program was utilized to conduct consensus clustering analysis, thereby determining the immunological subtypes. Through bioinformatics analysis, biomarkers associated with these immunological subtypes were identified, facilitating the development of a diagnostic predictive model. Potential targets for immunotherapy were identified by screening genes associated with overexpression, amplification, mutation, and prognosis. These findings were subsequently validated through a series of foundational experiments.

## 2. Materials and Methods

### 2.1. Data Acquisition and Preprocessing

The Cancer Genome Atlas (TCGA) database (https://portal.gdc.cancer.gov/) (accessed on 1 March 2023) was used to acquire the RNA sequencing information for 156 samples with TC. The details for 165 normal tissue samples from healthy individuals were supplied via the GTEx database (https://gtexportal.org/home/datasets) (accessed on 1 March 2023). The list of 1793 immune-related genes (IRGs) was collected from the IMMPORT database (https://immport.niaid.nih.gov/home) (accessed on 1 March 2023) (Supplementary Table S1). The progression-free interval (PFI) of TC was obtained and recommended for assessing the clinical outcome endpoints according to a study from Cell [16] (Supplementary Table S2).

### 2.2. PFI-Related IRGs Consensus Clustering and Validation

PFI-related IRGs were screened out through univariate Cox regression analysis from the list of IRGs. The “ConsensusClusterPlus” R package 4.0.0 [17] was utilized to separate immune subtypes in order to identify populations suitable for anti-tumor treatment based on the PFI-related IRGs’ expression. The best cluster numbers between k = 2 and 6 were identified, and 80 percent of the total samples were retrieved 1000 times to ensure the reliability and repeatability of the results. Calculating the “proportion of ambiguous clustering” (PAC) value can be implied to infer the ideal number of clusters. A low PAC value indicates a smooth middle segmentation and a low proportion of inaccurate allocations [18]. The PAC method, consensus clustering matrix, and the empirical cumulative distribution function (CDF) plot were used to determine the optimal cluster number. Then, principal component analysis (PCA) and t-Distributed Stochastic Neighbor Embedding (t-SNE) were performed to assess the stability and effectiveness of subtypes. Heatmaps of subtypes were drawn with the aid of the R software 4.0.0 package “pheatmap”.

### 2.3. Immune Infiltration Evaluation of Immune-Related TC Subtypes

The differences in the immune score, stromal score, ESTIMATE score, and tumor purity among the two subtypes were explored based on the ESTIMATE algorithm [19]. We performed single-sample Gene Set Enrichment Analysis (ssGSEA) [20] to calculate the proportion of immune cells. The expression level of immune checkpoint genes across the subtypes was compared, especially PD-L1, PDCD1, CTLA4, and LAG3. The difference between the subtypes was compared based on the Wilcoxon test. The tumor-immune dysfunction and exclusion (TIDE) scores of samples were collected from the TIDE website (https://tide.dfci.harvard.edu, accessed on 1 March 2023), which was developed by Liu et al. to forecast the effectiveness of immunotherapy [21].

### 2.4. WGCNA

The “WGCNA” package (R software 4.0.0) [22] was utilized to execute weighted gene co-expression network analysis (WGCNA) and derive gene co-expression modules from all IRGs. All the samples were used in the ensuing analysis as no outlier samples were found after the sample clustering. A correlation adjacency matrix was constructed adopting a soft-thresholding power of 5, following the scale-free network topology criterion. A topological overlap matrix (TOM) was created from the adjacency matrix to estimate the network connectedness. The TOM matrix was utilized as the foundation for constructing a hierarchical clustering tree, whose branches represented different gene modules. By merging and regrouping genes with similar expression patterns, resulting modules were created, each containing at least 30 genes. Module eigengenes (MEs) were estimated based on the resulting modules. The key modules that were notably associated with patient survival and subtypes were recognized by univariate Cox regression analysis. Module membership (MM) measures the association between genes as well as modules. The key genes were defined as genes with MM > 0.80 in the key modules (Supplementary Table S3). The “clusterProfiler” [23] (Version 4.2.2) package was utilized to perform Gene Ontology (GO) and Kyoto Encyclopedia of Genes and Genomes (KEGG) analyses, to identify the possible biological processes and signaling pathways of the key genes in the key modules.

### 2.5. Identification of Diagnostic Biomarkers for Subtypes

Based on the DEGs, key module genes, and key genes in the key modules, the hub genes were screened out. The expression trend of the hub genes between subtypes was presented by drawing heatmaps. The potential diagnostic biomarkers were defined as genes with consistent expression trends both between tumors and normal tissues and among subtypes.

### 2.6. Development and Assessment of the Diagnostic Model

The more important potential diagnostic biomarkers were further identified by the least (LASSO) [24] and support vector machine (SVM) [25] algorithms to develop a diagnostic model based on stepwise multiple logistic regression for evaluating the suitability of vaccination. The diagnostic model was presented through a nomogram plot and assessed by calibration curves, receiver operating characteristic (ROC) curves, Harrell’s concordance index (C-index), and decision curve analysis (DCA).

### 2.7. cBioPortal Analysis

The cBioPortal for Cancer Genomics (http://www.cbioportal.org) (accessed on 1 March 2023) was performed for the investigation, presentation, and analysis of genetic changes in TC as well as the acquisition of mutated genes (Supplementary Table S4) and amplified genes (Supplementary Table S4) in TC.

### 2.8. Gene Differential Expression Analysis and Survival Analysis

To minimize batch effects, the “Combat” algorithm from the “sva” package was utilized. The conditions of a |log2 Fold change (FC)| of more than 1 and a *p*-value of less than 0.05 were employed to identify the overexpressed gene (Supplementary Table S4), utilizing the R “DESeq2” (Version 3.50.1) package. The Kaplan–Meier method was utilized to perform a PFI-based survival analysis.

### 2.9. Correlation Between Antigen-Presenting Cells (APCs) and Potential Therapeutic Targets Expression

Utilizing the ssGSEA algorithm provided in the R package “GSVA” [26] and the markers of 24 immune cells obtained from a previous study [20], the immune infiltration level of the TC samples was calculated, and the correlation between APCs and potential therapeutic target expression was calculated using Spearman’s analysis.

### 2.10. Cell Culture and Reagents

Testicular carcinoma cell lines NTERA-2 were purchased from ATCC. All cells were cultured with Dulbecco’s Modified Eagle’s Medium (DMEM; Gibco Company, Grand Island, NY, USA) with 10% fetal bovine serum (FBS; Gibco, Thermo Fisher Scientific, Waltham, MA, USA, Cat# 26140079) and 1% penicillin/streptomycin (Invitrogen, Carlsbad, CA, USA, Cat# 10378016) and at 37 °C with 5% CO_2_.

### 2.11. Transient Transfection

NTERA-2 cells were grown to 60% confluence in 6-well plates and transfected with 600 pmol of siRNA. At 48 h after transfection, the cells were collected, and total protein lysates were prepared for Western blot analysis. The siRNAs (Shanghai GenePharma, Shanghai, China, Cat# stQ0007602-1) were as follows:

GRK5 siRNA-1 (sense), 5′-GGACCAUAGACAGAGAUUATT-3′, (antisense),5′-UAAUCUCUGUCUAUGGUCCTT-3′;
GRK5 siRNA-2 (sense), 5′-CCCUCAAUGAGAAGCAGAUTT-3′, (antisense),5′-AUCUGCUUCUCAUUGAGGGTT-3′;
GRK5 siRNA-3 (sense), 5′-GAACGUGUUUGGACCUAAUTT-3′, (antisense),5′-AUUAGGUCCAAACACGUUCTT-3′;
GRK5 siRNA-4 (sense), 5′-CAUCCUGUUAGAUGAUUAUTT-3′, (antisense),5′-AUAAUCAUCUAACAGGAUGTT-3′;
WNT11 siRNA-1 (sense), 5′-GCCUCUCUGGAAAUGAAGUTT-3′, (antisense),5′-ACUUCAUUUCCAGAGAGGCTT-3′;
WNT11 siRNA-2 (sense), 5′-CAGGAUCCCAAGCCAAUAATT-3′, (antisense),5′-UUAUUGGCUUGGGAUCCUGTT-3′;
WNT11 siRNA-3 (sense), 5′-CAACAAGACAUCCAACGGATT-3′, (antisense),5′-UCCGUUGGAUGUCUUGUUGTT-3′;
WNT11 siRNA-4 (sense), 5′-GAACUCGUCUAUCUGCAGATT-3′, (antisense),5′-UCUGCAGAUAGACGAGUUCTT-3′;
FAM181B siRNA-1 (sense), 5′-GCGAUCUACUCAGCUUCAUTT-3′, (antisense),5′-AUGAAGCUGAGUAGAUCGCTT-3′;
FAM181B siRNA-2 (sense), 5′-GUGGGAAACCUACUGUACCTT-3′, (antisense),5′-GGUACAGUAGGUUUCCCACTT-3′;
FAM181B siRNA-3 (sense), 5′-CAGGUGUCCUACGAUUACATT-3′, (antisense),5′-UGUAAUCGUAGGACACCUGTT-3′;
FAM181B siRNA-4 (sense) ,5′-GUUUCGAGGACGAUGAGACTT-3′, (antisense),5′-GUCUCAUCGUCCUCGAAACTT-3′;
FSCN1 siRNA-1 (sense), 5′-CCUCAGGUCAACAUCUACATT-3′, (antisense),5′-UGUAGAUGUUGACCUGAGGTT-3′;
FSCN1 siRNA-2 (sense), 5′-GCUGCUACUUUGACAUCGATT-3′, (antisense),5′-UCGAUGUCAAAGUAGCAGCTT-3′;
FSCN1 siRNA-3 (sense), 5′-GUGGACUUCUUCUUCGAGUTT-3′, (antisense),5′-ACUCGAAGAAGAAGUCCACTT-3′;
FSCN1 siRNA-4 (sense), 5′-GCCUACAACAUCAAAGACUTT-3′, (antisense),5′-AGUCUUUGAUGUUGUAGGCTT-3′;
ECHS1 siRNA-1 (sense), 5′-CCUUCGCCUCGGGUGCUAATT-3′, (antisense),5′-UUAGCACCCGAGGCGAAGGTT-3′.
ECHS1 siRNA-2 (sense), 5′-GACUGUUACUCCAGCAAGUTT-3′, (antisense),5′-ACUUGCUGGAGUAACAGUCTT-3′.
ECHS1 siRNA-3 (sense), 5′-CCUCAAUGCACUUUGCGAUTT-3′, (antisense),5′-AUCGCAAAGUGCAUUGAGGTT-3′.
ECHS1 siRNA-4 (sense), 5′-GGUCUUGUCAGCAAGAUUUTT-3′, (antisense),5′-AAAUCUUGCUGACAAGACCTT-3′.

We also used si-NC for transfection control.

### 2.12. Transfection and Generation of Stable Cell Lines

For the GRK5 knockdown experiment, we transfected NTERA-2 cells with plasmids containing a short hairpin RNA directed against the human GRK5 gene or the negative control (Shanghai GenePharma, Shanghai, China, Cat# shQ0008501-6). The overexpressed vector was ordered from OBiO (Shanghai, China). Lentivirus and Polybrene with a final concentration of 5 μg/mL were added to cultured NTERA-2 cells for 24 h. After that, the cells were cultured in fresh medium for another 24 h. The stable transfected cells were screened with a culture medium containing purinomycin. The transfection efficacy was determined by Western blot analysis.

The sequences were as follows:

LV3-KD-GRK5, 5′-CCAGTTGTAACCACCGAATAA-3′

LV3-OE-GRK5, 5′-GCCTGGTTCTGACCATTATGA-3′

LV-3NC, 5′-TTCTCCGAACGTGTCACGT-3′

### 2.13. Western Blot Analysis

Proteins were isolated from these transfected cells by RIPA Lysis Buffer (Beyotime, Shanghai, China). The concentration of proteins was quantified using a BCA Protein Assay Kit (Beyotime, Shanghai, China). Extracted proteins were separated by SDS-PAGE electrophoresis and transferred onto PVDF. The membrane was blocked with 5% non-fat milk in TBST, and then probed overnight at 4 °C with primary antibodies:WNT11 (1:1000, Invitrogen, USA), FAM181B (1:1000, Invitrogen, USA), GRK5 (1:1000, Proteintech, Wuhan, China, Cat No. 17032-1-AP), FSCN1 (1:1000, Proteintech, China), ECHS1 (1:1000, Proteintech, China). After washing three times, membranes were incubated with the secondary antibody for 1 h at room temperature and re-washed 3 times. Then membranes were exposed using the ECL system (Thermo Fisher Scientific, Waltham, MA, USA, Cat# 34580).

### 2.14. Cell Proliferation Assay

Cell proliferation was measured by CCK-8 assay. Pretreated cells were counted and seeded into 96-well plates at a density of 5000 cells/well in a volume of 100 µL. After 24, 48, 72, and 96 h, 10 µL CCK8 solution (Catalog No: C0005, TargetMoI) was added to each well, which were incubated for 2 h at 37 °C and 5% CO_2_. The optical absorbance was measured at a wavelength of 450 nm.

### 2.15. Wound-Healing Assay

Cells from each group were seeded into the 6-well plates with a confluence of approximately 95%. Then, 200 µL pipette tips were used to make a symmetrical wound. After washing with PBS, the cells were incubated with non-serum DMEM medium for 12, 24, and 48 h at 37 °C and 5% CO_2_. Migration photos were taken 12, 24, and 48 h after wound drawing. Then, Image J software 1.8.0 was used to measure the wound distance of each group at 40× magnification and to calculate the wound-healing rate.

### 2.16. Transwell Assay

Transwell migration was measured using 8 um Transwell chambers (Corning, NY, USA, Cat# 353097). Then, 2 × 10^4^ cells with non-serum medium were inoculated into the 24-well upper chambers, and 600 µL of 15% FBS medium was added to the lower chamber. The cells were incubated for 24 h in a migration test. The cells in the chamber were fixed with 4% paraformaldehyde, incubated, and stained with 0.1% crystal violet. The cells invading in each of the three random fields were counted under a 100× microscope. The results were analyzed using Image J software.

### 2.17. Apoptosis Assay

Cells were collected 48 h after transfection and were treated with 0.1% hydrogen peroxide for 8 h, and the rate of apoptosis in each group was detected using the Alexa Fluor 488 Annexin V/Dead Cell Apoptosis Kit (ThermoFisher scientific), according to the manufacturer’s instructions. Treated cells were harvested and double-stained with Annexin V-FITC and propidium iodide (PI) in the absence of light.

Flow cytometry analysis was performed on a BD FACSCanto II system (BD Biosciences, San Jose, CA, USA). The FITC signal was detected using 488 nm excitation and 530 nm emission (green fluorescence channel). The PI signal was detected using 488 nm excitation and 620 nm emission (red fluorescence channel). Cells were classified as living cells, early apoptotic cells, advanced apoptotic cells, and necrotic cells. At least 10,000 events were recorded for each replicate and the apoptotic rate indicates the percentage of cells at quadrant Annexin V-FITC + and PI − or +. The results were analyzed using FlowJo software 10.8.1.

### 2.18. In Vitro T-Cell Killing Assay

Human PBMCs were isolated, extracted, and subsequently seeded at a density of 1 × 10^6^ cells in 1640 medium. Activation was induced by supplementing the culture with cytokine H-IL-2 (10 ng/mL), CD3 (2 μg/mL), and CD28 (2 μg/mL). The experimental setup included four groups: a blank group (medium only); a control group (untreated NTERA-2 cells); the sh-NC group; and the sh-GRK5 group. Each group was co-incubated with PBMCs for 0, 4, 12, and 24 h. Following incubation, 10 μL of CCK8 was added to each well, and the plates were incubated for an additional 2 h. Absorbance was then measured at 450 nm to evaluate cell viability.

Then, they were divided into the following groups: a blank group (medium only); the unactivated PBMC group; the NC group; the KD group (NTERA-2 with GRK5 knockdown); the GRK5-IN-2 group (NTERA-2 cells treated with GRK5 inhibitor GRK5-IN-2 [Catalog No. HY-136561, MedChemExpress]); and the OE group (NTERA-2 with GRK5 overexpressed). Each group was co-incubated with PBMC. After incubation for 24-h, cell-free supernatants were obtained by centrifuging. Aliquots of supernatants were frozen at −20 °C, pending assay. The concentration of TNF-α, IFN-γ, IL-12 and IL-6 in the supernatant of co-cultured and monocultured groups was measured using an ELISA kit (DAKEWE, China) following the manufacturer’s instruction. The relative optical density was measured at 450 nm and 610 nm.

### 2.19. Statistical Analyses

All data visualization and statistical analysis were accomplished by R software (Version 4.1.2). A *p*-value <0.05 was considered statistically significant. All the experiments were performed at least three independent times, and processed using GraphPad Prism 10.1.2 software. Data are represented as mean ± standard deviation (SD), and error bars also indicate SD. The *p*-values were calculated by either unpaired or paired two-tailed Student’s *t* test, * *p* < 0.05, ** *p* < 0.01, *** *p* < 0.001, and **** *p* < 0.0001.

## 3. Results

### 3.1. Identification of Immune-Related TC Subtypes

**Figure 1:**
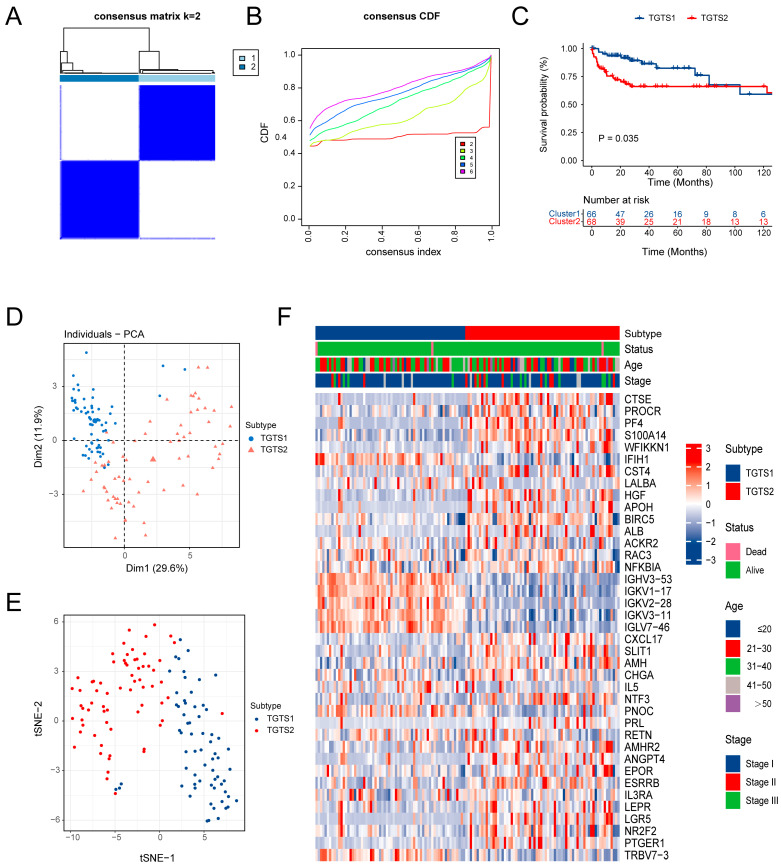
Identification of Immune-Related TC Subtypes. (**A**) Consensus matrix of samples in TCGA-TC, for k = 2. (**B**) The cumulative distribution function curves, k = 2 to 6. (**C**) Survival analysis of the two subtypes in the TCGA-TC cohort. (**D**) The plot of principal component analysis (PCA). (**E**) The plot of t-distributed stochastic neighbor embedding. (**F**) Heatmap presenting the expression profiles of prognostic-related IRGs and clinicopathologic features in two subtypes.

Identifying immunotypes to reflect the immunological microenvironment and immune status within tumors enables the differentiation of patients who may benefit from anti-tumor treatments. In this study, all 1793 immune-related genes (IRGs) from the TCGA-TC cohort were analyzed using univariate Cox regression to identify the most significant prognosis-related IRGs (*p* < 0.05), which were then subjected to consensus clustering analysis. Two distinct immune-related subtypes were identified: testicular germ tumor subtype 1 (TGTS1) and testicular germ tumor subtype 2 (TGTS2), based on the consensus clustering matrix (Figure 1A), the CDF plot (Figure 1B), and the proportion of ambiguous clustering (PAC) method. TGTS1 was associated with significantly better prognoses compared to TGTS2 (Figure 1C). The distributions of TGTS1 and TGTS2 were visualized using PAC (Figure 1D) and t-SNE (Figure 1E) plots, demonstrating the robustness and efficacy of the clustering analysis. Specifically, TGTS2 exhibited elevated expression levels of the majority of prognostic IRGs (Figure 1F).

### 3.2. Association Between Immune Infiltration Characteristics Between the Two Immune Subtypes

TGTS1 and TGTS2 exhibited distinct immune infiltration characteristics, as illustrated in Supplementary Figure S1. TGTS1 demonstrated significantly higher immune and ESTIMATE scores (*p* < 0.05) (Supplementary Figure S1A) and lower tumor purity (*p* < 0.05) (Supplementary Figure S1B), indicating a greater presence of immune-related components in TGTS1. According to the ssGSEA findings, TGTS1 samples contained a more abundant array of immune cells compared to TGTS2 (Supplementary Figure S1C). The expression levels of immune checkpoint genes were also compared (Supplementary Figure S1D), revealing that TGTS1 exhibited higher expression levels of PD-L1, PDCD1, CTLA4, and LAG3 genes than TGTS2 (Supplementary Figure S1E–H). The TIDE algorithm, known for its superior predictability of immunotherapy responses compared to PD-L1 levels or mutation load alone, was applied. Our analysis revealed higher TIDE scores in TGTS2, suggesting a correlation with less effective immune checkpoint inhibition.

### 3.3. Identification of Critical Immune-Related Gene Modules

**Figure 2:**
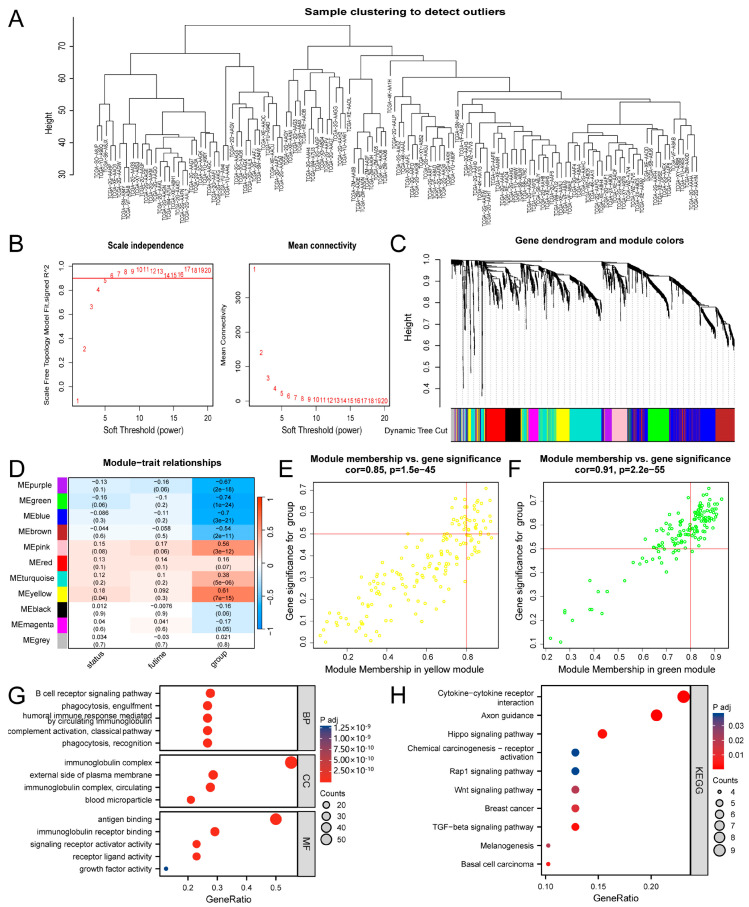
Identification of critical immune-related gene modules. (**A**–**C**) Co-expression network of immune-associated genes. (**D**) Correlations between modules and survival and subtype. (**E**,**F**) The key genes with MM > 0.80 in the “yellow” module (**E**) and the “green” module (**F**). (**G**) GO analysis of the key genes. (**H**) KEGG analysis of the key genes.

All IRGs were utilized to weighted gene co-expression network analysis (WGCNA) to identify key subtype markers that could aid in determining populations suitable for vaccination. All samples were included in the subsequent analysis as no outliers were detected after sample clustering (Figure 2A). The soft threshold was set at 5 in the scale-free network (Figure 2B). After conversion to the TOM, 11 modules were identified by merging and recombining genes with similar expression patterns, with each module containing a minimum of 30 genes (Figure 2C). The “yellow” and “green” modules exhibited a significant association with patient survival and subtypes (Figure 2D). Key genes with MM > 0.80 in the “yellow” and “green” modules (Figure 2E,F) were selected for further analysis (Supplementary Table S3). GO and KEGG analyses indicated that the key genes in these modules were closely linked to immune activation and classical pathways (Figure 2G,H). These key genes demonstrated potential for distinguishing patient survival status and immune subtypes, thereby assisting in identifying candidates for immunotherapy.

### 3.4. Identification of Potential Diagnostic Biomarkers

Based on DEGs, key module genes, and key genes within these modules, 33 up-regulated hub genes and 70 down-regulated hub genes distinguishing TGTS1 from TGTS2 were identified (Supplementary Figure S2A–D). Among these, CTSE, LGR5, CLDN4, CRABP2, BMP7, BMP4, STC1, ESM1, BMP2, HNF4A, PROC, SEMA3E, SEMA5B, NTS, DKK1, FGF17, JAG1, VTN, and SSTR2 were up-regulated hub genes, exhibiting higher expression levels in tumor samples compared to normal samples (Supplementary Figure S2E). No down-regulated hub genes showed consistent expression trends across both tumor versus normal tissues and among subtypes (Supplementary Figure S2F). These 19 up-regulated hub genes present potential as diagnostic biomarkers for distinguishing TGTS1 from TGTS2.

### 3.5. Development and Assessment of a Diagnostic Model

**Figure 3:**
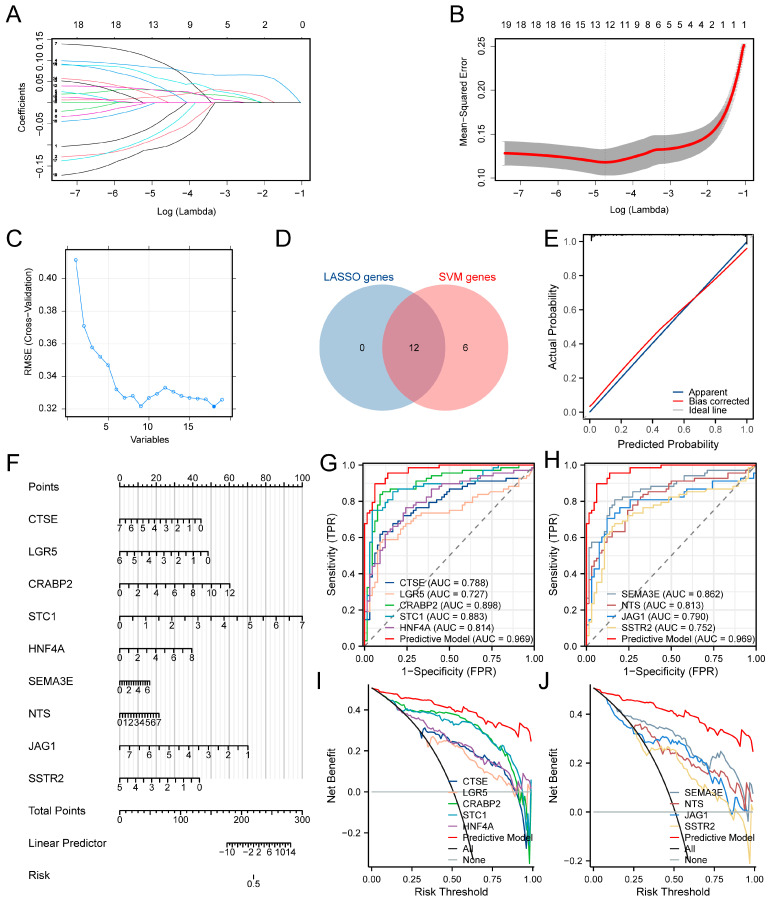
Construction and assessment of a diagnostic model presented through a nomogram plot. (**A**,**B**) LASSO COX algorithms of potential diagnostic biomarkers. (**C**) SVM algorithm of potential diagnostic biomarkers. (**D**) Venn plot shows the overlapping potential diagnostic biomarkers based on LASSO and SVM algorithms. (**E**) A nomogram plot based on the 12 more significant diagnostic biomarkers. (**F**) The calibration curve for internal validation of the nomogram. (**G**,**H**) The ROC curves of the nomogram and 12 diagnostic biomarkers. (**I**,**J**) DCA of the nomogram and 12 diagnostic biomarkers.

Using LASSO and SVM algorithms, we further explored significant potential diagnostic biomarkers. Twelve genes (CTSE, LGR5, CLDN4, CRABP2, STC1, ESM1, HNF4A, PROC, SEMA3E, NTS, JAG1, and SSTR2) were identified based on the minimum partial likelihood deviance (Figure 3A,B). According to the SVM algorithm, 18 genes, excluding DKK1, were deemed relatively more important (Figure 3C). Both LASSO and SVM algorithms highlighted twelve genes as relatively more significant (Figure 3D). Subsequent stepwise multiple logistic regression identified nine key genes (CTSE, LGR5, CRABP2, STC1, HNF4A, SEMA3E, NTS, JAG1, and SSTR2) based on the minimum Akaike information criterion. A diagnostic model was constructed using these nine genes and presented through a nomogram plot (Figure 3F), demonstrating strong predictive performance. Calibration curves showed a high degree of concordance between predicted and actual survival probabilities (Figure 3E). The model’s area under the curve (AUC) value was highest at 0.969 (Figure 3G,H), with a C-index of 0.969 and a 95% confidence interval of 0.945–0.993. The nomogram provided a significant net benefit over individual genes, as demonstrated by DCA (Figure 3I,J). This diagnostic model is poised to assist clinicians in more accurately identifying immune subtypes in TC patients.

### 3.6. Identification of Potential Therapeutic Targets

To identify potential therapeutic targets for the development of tailored anti-tumor treatments, we analyzed genome copy number alterations in the TCGA-TC dataset (Supplementary Figure S3A). This analysis revealed 3256 genes exhibiting copy number amplification (amplified rate > 1%) (Supplementary Table S4). Differential analysis identified 10,027 DEGs (Supplementary Figure S3B), among which 5392 were overexpressed (Supplementary Table S4). By assessing mutation counts and altered genome fractions, we identified 5665 mutant genes with the potential to encode tumor-specific antigens (Supplementary Figure S3C,D). The top 10 genes with the highest average frequency of mutational counts and altered genome fractions were identified through an investigation of genome alterations (Supplementary Figure S3E,F). Notably, KRAS, SCNN1A, ERC1, A2M, CD9, VWF, and ZNF384 emerged as the most frequently mutated genes in both mutation count and altered genome fraction groups (Supplementary Figure S3E,F). Combining highly amplified, overexpressed, and frequently mutated genes, we identified 203 overlapping genes as potential candidate therapeutic targets for TC (Supplementary Table S4).

### 3.7. Identification of the Prognosis-Related TC Therapeutic Targets

**Figure 4:**
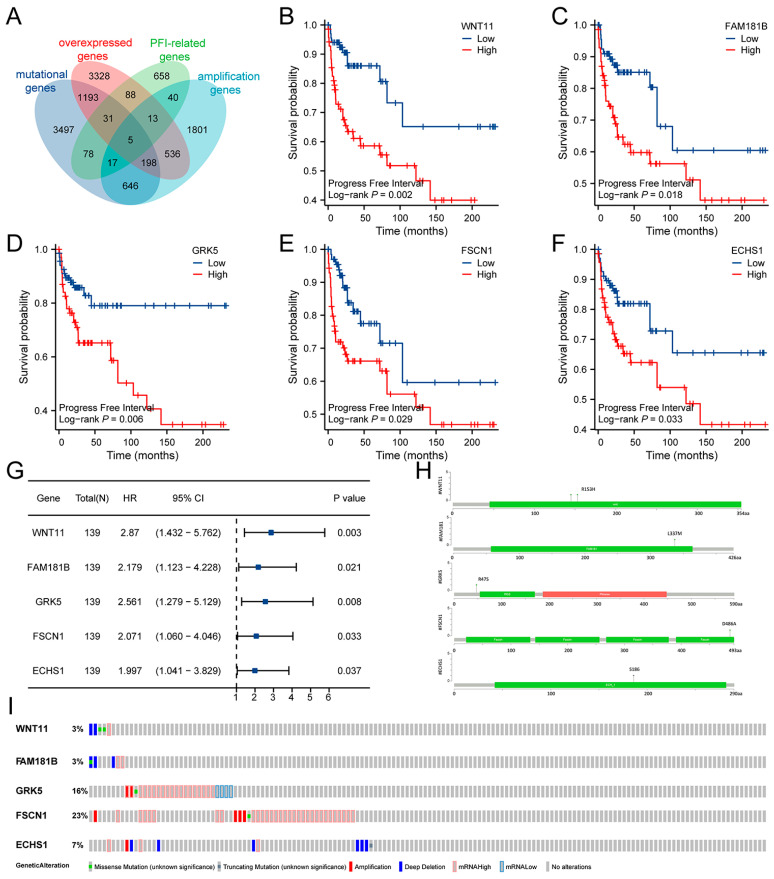
Identification of tumor-specific tumor therapeutic targets related to the prognosis of TC. (**A**) Selection of the prognostic-related tumor therapeutic targets based on mutational, amplification, PFI-related, and overexpressed genes. (**B**–**F**) Kaplan–Meier survival curve analysis for WNT11 (**B**), FAM181B (**C**), GRK5 (**D**), FSCN1 (**E**), and ECHS1 (**F**) in the TCGA-TC cohort. The log-rank test was used to determine the statistical significance of the differences, and *p* < 0.05 was considered significant. (**G**) The univariable COX analysis of five tumor therapeutic targets related to the prognosis of TC. (**H**,**I**) The OncoPrint tab summarizes genomic alterations across the TCGA-TC cohort.

PFI-related genes in TC were identified through survival analysis. Five genes (WNT11, FAM181B, GRK5, FSCN1, and ECHS1) emerged as prognostic-related therapeutic targets based on four gene sets: amplification, overexpression, mutation, and PFI-related genes (Figure 4A). Survival curves indicated that individuals with high expression levels of WNT11, FAM181B, GRK5, FSCN1, and ECHS1 had worse prognoses (Figure 4B–F). Univariate COX regression analysis further confirmed that all five genes (WNT11, FAM181B, GRK5, FSCN1, and ECHS1) were significant risk indicators for TC (Figure 4G). The OncoPrint tab effectively summarized genomic alterations across the TCGA-TC cohort (Figure 4H,I).

### 3.8. Correlation Between Prognosis-Related TC Therapeutic Targets and APCs

APCs are essential for recognizing antigens and triggering immunological reactions. Thus, the correlation between five candidate TC therapeutic targets and APCs was further investigated. The expression of WNT11, FAM181B, GRK5, FSCN1, and ECHS1 presented a positive association with the infiltration level of B cells (Supplementary Figure S4A,D,G,J,M). Overall, there was a positive correlation between the level of any antigen’s expression with the infiltration of at least one type of APC (Supplementary Figure S4A–O). These findings demonstrated that APCs might identify and process WNT11, FAM181B, GRK5, FSCN1, and ECHS1 for the activation of immunological responses. Meanwhile, WNT11, FAM181B, and FSCN1 are differentially expressed in different subtypes, demonstrating that they might be potential therapeutic targets in TGTS2.

### 3.9. GRK5 Silencing Significantly Inhibits Malignancy

**Figure 5:**
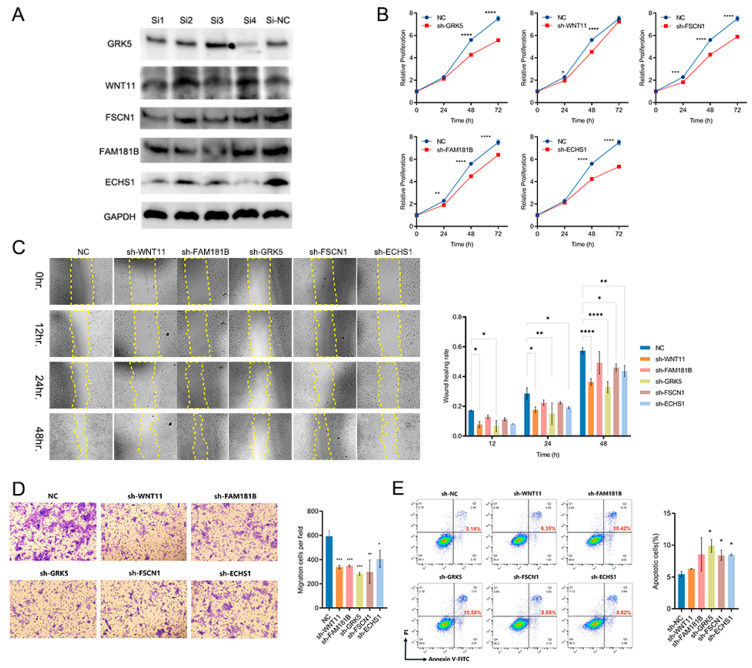
Cellular Functional Assays. (**A**) WB analysis was conducted to confirm the knockdown efficiency of each Si-RNA. The original WB images are provided in the Supplementary Materials. (**B**) Cell Counting Kit-8 (CCK8) assay was performed, where * denotes a statistically significant difference. (**C**) A wound-healing assay was conducted, with * indicating statistical significance. (**D**) Transwell migration assay was employed, and * denotes a statistically significant difference when compared to the negative control (NC) group. (**E**) Apoptosis was assessed, with red numerals indicating the proportion of apoptotic cells. * denotes a statistically significant difference relative to the NC group.* *p* < 0.05, ** *p* < 0.01, *** *p* < 0.001, and **** *p* < 0.0001.

Using a bioinformatics platform, four highly specific small interfering RNAs (siRNAs) were designed per target gene, incorporating cross-species conservation analysis and off-target effect prediction to ensure silencing specificity. These siRNAs were transfected into the NTERA-2 cell line, with three biological replicates per siRNA and a negative control siRNA (NC) group. Western blot analysis confirmed that at least one effective siRNA was identified for each of the five candidate genes (Figure 5A). Cell proliferation was assessed using the CCK-8 assay in control and target gene-knockdown cells. Absorbance was measured at 0, 24, 48, and 72 h after plating. The results demonstrated significantly reduced growth rates in all five candidate target gene-knockdown groups compared to the control. Notably, GRK5 and ECHS1 exhibited the most pronounced inhibitory effects, with relative proliferation decreasing by 1.932 (*p* < 0.0001) and 2.171 (*p* < 0.0001), respectively, compared to the NC group after 72 h (Figure 5B). Further, wound-healing assays demonstrated a substantial reduction in the migratory capacity of NTERA-2 cells following the knockdown of WNT11, GRK5, FSCN1, and ECHS1, with GRK5 exerting the strongest effect (healing percentage: 32.95%, *p* < 0.0001) (Figure 5C). Consistently, transwell assays corroborated these findings, showing a marked decrease in invasion capacity after knockdown, with GRK5 again displaying the most significant impact (migration cells per field: 283, compared to 593 in the NC group, *p* = 0.0004) (Figure 5D). To further investigate the effects of gene silencing on tumor cell apoptosis, we performed flow cytometry. This analysis revealed that GRK5 knockdown led to a significant increase in the proportion of early and late apoptotic cells, rising from 5.14% to 10.58% (*p* = 0.0286), while the other targets demonstrated comparatively weaker effects (Figure 5E).

### 3.10. Inhibition of GRK5 Significantly Activates Anti-Tumor Immunity

**Figure 6:**
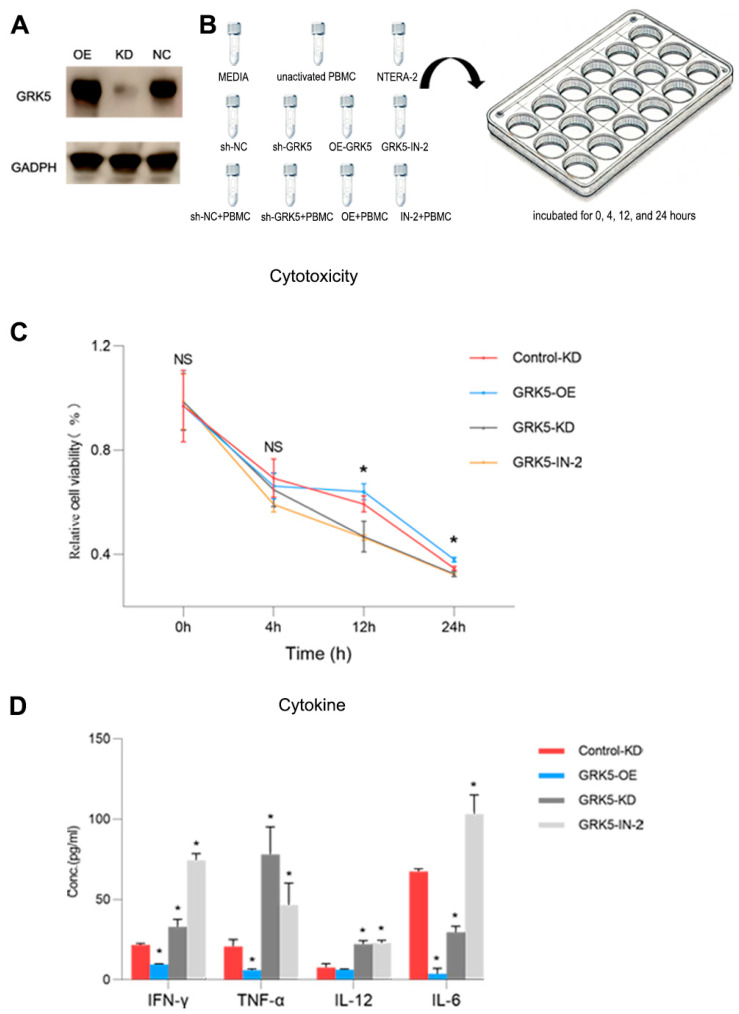
In vitro T-cell killing assay. (**A**) Western Blot Analysis of Stable GRK5-KD and GRK5-OE Cell Lines. (**B**) depict schematic diagrams of the experimental groups. The line graph (**C**) illustrates the changes in PBMC cytotoxicity over the incubation period. PBMC cytotoxicity is calculated as the cell viability of the KD + PBMC group relative to that of the KD group. The formula for calculating cell viability is defined as: Cell viability = (Experimental group − Blank)/(NTERA2 − Blank). The bar chart (**D**) illustrates the concentration of cytokines produced by PBMCs. * *p* < 0.05; ns, not significant.

To better investigate the role of GRK5, we established stable cell lines with GRK5 overexpression (OE) and knockdown (KD) for functional analyses (Figure 6A). The results of the T-cell cytotoxicity assay indicated that, after accounting for the confounding effects of GRK5 inhibition or activation on cell proliferation (Figure 6B), the survival rates of cells in the GR-IN-2 group (which competitively binds to the GRK5 ligand) (12 h: 46.5%; 24 h: 32.3%) and the knockdown group (12 h: 46.8%; 24 h: 32.5%) were significantly lower than those in the control group (12 h: 59.3%; 24 h: 34.7%) following 12 h and 24 h of co-culture with PBMCs (Figure 6C). Conversely, the survival rate in the OE group (12 h: 64.0%; 24 h: 38.0%) was higher than that of the control group (Figure 6B). Furthermore, we quantitatively analyzed the cytokine concentrations in the supernatant of the PBMC-tumor cell co-culture system. Specifically, GRK5 overexpression markedly suppressed IFN-γ (9.59 ± [0.34]) and IL-6 (3.80 ± [3.37]) levels compared to the control group (Control-KD: IFN-γ = 21.67 ± [0.90]; IL-6 = 67.42 ± [1.65]). Conversely, GRK5 knockdown elicited a modest increase in IFN-γ (32.99 ± [4.68]), a pronounced elevation of TNF-α (78.02 ± [17.12] vs. Control-KD: 20.69 ± [4.36]), and moderate upregulation of both IL-12 (22.14 ± [2.19]) and IL-6 (29.59 ± [3.71]). GRK5-IN-2 induced a robust surge in IFN-γ (74.39 ± [4.18]), IL-6 (103.20 ± [11.84]), and IL-12 (22.90 ± [1.69]), accompanied by a moderate rise in TNF-α (46.58 ± [13.63]) (Figure 6D).

## 4. Discussion

Identifying the appropriate population for specific immunotherapy represents a significant challenge in the field of cancer treatment. Utilizing advanced bioinformatics techniques, we have delineated the immunological subtypes of TC and investigated the immunological disparities between these subtypes. Patients with TC were classified into two distinct immune-related subtypes, namely TGTS1 and TGTS2, each characterized by unique immune statuses and molecular features. TGTS2, in contrast to TGTS1, displays an immunologically cold phenotype, marked by lower levels of immune infiltration and diminished expression of immune-related genes. By targeting specific immunotherapy pathways, it is feasible to convert these “cold” tumors into “hot” tumors, thereby eliciting a robust immune response against the malignancy [27,28]. Our analysis revealed that TGTS1 exhibited higher expression levels of immune checkpoint genes, particularly PD-L1, PDCD1, CTLA4, and LAG3, compared to TGTS2. This suggests that individuals classified under TGTS2 may be optimal candidates for specific anti-tumor immunotherapies, whereas TGTS1 individuals may derive greater benefit from immune checkpoint inhibitors [29].

To further assist clinicians in identifying suitable candidates for tumor vaccines, we employed machine learning algorithms to discover potential biomarkers and develop predictive models. Through WGCNA, we identified key modules and genes significantly associated with patient survival and subtypes. Leveraging LASSO and SVM algorithms, we refined the search for significant diagnostic biomarkers. Subsequent stepwise multiple logistic regression highlighted nine genes (CTSE, LGR5, CRABP2, STC1, HNF4A, SEMA3E, NTS, JAG1, and SSTR2), which were selected based on the minimum Akaike information criterion (AIC) for constructing a robust diagnostic model. The performance of this model was validated through calibration curves, ROC curves, C-index, and DCA, all of which demonstrated its robustness. As a precise identification tool, this diagnostic model holds promise for accurately determining the appropriate population for specific immunotherapies. Candidate therapeutic targets, including WNT11, FAM181B, GRK5, FSCN1, and ECHS1, have been identified as overexpressed, amplified, mutated, associated with prognosis, and related to antigen-presenting cells. However, our conclusions are currently supported solely by bioinformatics analysis and other methodologies, underscoring the need for further experimental validation.

To further validate the role of the aforementioned target genes, we conducted a series of molecular biology experiments. During the initial preparation of the target gene-silenced cell lines, four distinct knockdown sites were employed for each gene to ensure optimal silencing efficacy. Subsequent functional assays revealed that among the five target genes, GRK5 had the most pronounced impact on cancer cell proliferation, invasion, metastasis, and immortality. This led us to hypothesize that GRK5 functions as an oncogene in TC. Given this, we also sought to investigate the effect of GRK5 on immunogenicity. However, the knockdown of GRK5 inherently inhibits cell proliferation. To account for this confounding factor, we performed a self-control experiment, comparing T-cell co-culture with single culture conditions to isolate the gene’s background influence on proliferation. The results indicated that GRK5 facilitates immune evasion in TC cells. The selection of specific cytokines (TNF-α, IFN-γ, IL-12, and IL-6) for assessing T-cell-mediated immune activation was guided by their established roles in anti-tumor immunity. IFN-γ is a master regulator of MHC class I/II expression and tumor antigen presentation, while TNF-α directly induces cancer cell apoptosis through caspase activation [9,20]. IL-12 promotes the differentiation of naïve T-cells into cytotoxic CD8^+^ T effectors and synergizes with IFN-γ to enhance tumor cell killing [21]. Although IL-6 may support tumor progression in chronic settings, its acute elevation reflects early T-cell activation and inflammatory responses [30]. This cytokine panel thus captures key facets of immunogenic conversion: cytotoxic effector function (IFN-γ/TNF-α), T-cell priming (IL-12), and inflammatory milieu modulation (IL-6). Consequently, we propose that targeting GRK5 could not only attenuate the proliferation and spread of TC but also enhance the tumor’s immune response, making it a potentially effective therapeutic target for the development of anti-tumor immunotherapy in TC.

Beyond its established role in GPCR desensitization, GRK5 may orchestrate immune evasion through multiple mechanisms. First, as a regulator of chemokine receptors (e.g., CXCR4 and CCR5) [30], GRK5 could impair T-cell chemotaxis by internalizing these receptors, limiting lymphocyte infiltration into tumors—a phenomenon observed in our immunologically ‘cold’ TGTS2 subtype. Second, our data demonstrate that GRK5 suppression substantially elevates TNF-α and IFN-γ (Figure 6D), cytokines that induce MHC class I/II expression [9,20]; this suggests GRK5 may dampen tumor immunogenicity by suppressing antigen presentation machinery. Third, emerging evidence indicates that GRK5 phosphorylates PD-1’s intracellular domain in T-cells [31], potentially stabilizing this immune checkpoint. Notably, in prostate cancer models, GRK5 knockdown reduced PD-L1 expression by inhibiting STAT3 phosphorylation [32]. While these mechanisms require validation in TC, our findings that GRK5 inhibition (i) enhances APC-mediated immune responses (Supplementary Figure S4) and (ii) synergizes with T-cell cytotoxicity (Figure 6C) position it as a master regulator of tumor-immune crosstalk.

To assess the current understanding of the relationship between GRK5 and TC, we conducted an extensive literature review. However, our search yielded no studies specifically addressing GRK5 in the context of TC. Consequently, we broadened our scope to include pan-cancer analyses, identifying studies that have explored the role of GRK5 in prostate cancer [33,34], breast cancer [35,36], and kidney cancer [37]. Notably, aside from two high-quality articles on prostate cancer, the majority of the literature we reviewed was of moderate quality and published in journals of limited impact. Given the relevance of these two prostate cancer studies, we provide a brief review of their findings. Both studies identify GRK5 as an oncogene in prostate cancer. The study published in Cancer Research posits that GRK5 facilitates prostate cancer invasion through the regulation of actin dynamics [33], while the Journal of Urology article suggests that GRK5 promotes prostate cancer proliferation by modulating the cell cycle and tumor suppressor genes [34]. These findings align with our own observations and offer valuable insights for future investigations into the pathway mechanisms underlying GRK5’s role in cancer.

## 5. Conclusions

Through bioinformatics screening and experimental validation, we have demonstrated that GRK5 promotes the proliferation, migration, and invasion of testicular cancer, while simultaneously suppressing tumor immunity.

## Data Availability

The datasets analyzed during this study are available in the The Cancer Genome Atlas (TCGA) (https://tcga-data.nci.nih.gov/tcga/, accessed on 1 February 2023) and GTEx database (https://gtexportal.org/home/datasets, accessed on 1 February 2023). The immune-related genes were recorded the IMMPORT database (https://immport.niaid.nih.gov/home, accessed on 1 February 2023). The raw data supporting this publication are available from the Figshare repository (DOI: 10.6084/m9.figshare.27161856).

## Supplementary Materials

The following supporting information can be downloaded at: https://www.mdpi.com/article/10.3390/biomedicines13071775/s1, Figure S1: Comparison of immune infiltration characteristics between subtypes; Figure S2: Identification of potential diagnostic biomarkers; Figure S3: Identification of candidate tumor therapeutic targets of TC; Figure S4: Correlation between candidate tumor therapeutic targets and antigen-presenting cells; Table S1: The list of 1793 immune-related genes; Table S2: The progression-free interval (PFI) of TC was obtained and recommended for assessing the clinical outcome endpoints according to a study from Cell; Table S3: List of key genes with MM > 0.80 in key module; Table S4: Mutated genes and amplified genes in TC.

## Abbreviations

The following abbreviations are used in this manuscript:

TC	testicular cancer
TCGA	The Cancer Genome Atlas
WGCNA	gene co-expression network analysis
SVM	support vector machine
LASSO	the least absolute shrinkage and selection operator regression
MsigDB	Molecular Signatures Database
t-SNE	t-Distributed Stochastic Neighbor Embedding
PCA	principal component analysis
IRGs	immune-related genes
APCs	antigen-presenting cells
TGTS1	testicular germ tumor subtype 1
TGTS2	testicular germ tumor subtype 2
ssGSEA	single-sample Gene Set Enrichment Analysis
IPS	immunophenoscore
TIDE	tumor-immune dysfunction and exclusion
GO	Gene Ontology
KEGG	Kyoto Encyclopedia of Genes and Genomes
GSEA	Gene set enrichment analysis
PPI	Protein-protein interaction
TOM	topological overlap matrix
ROC	receiver operating characteristic
DCA	decision curve analysis
CDF	cumulative distribution function
AUC	area under the curve
