# Supplementary material for: GRK5 as a Novel Therapeutic Target for Immune Evasion in Testicular Cancer: Insights from Multi-Omics Analysis and Immunotherapeutic Validation
Source: Biomedicines. 2025 Jul 21;13(7):1775. doi: 10.3390/biomedicines13071775 (PMC12292343; doi:10.3390/biomedicines13071775)
Supplement: Supplementary file 1 [file biomedicines-13-01775-s001.zip › Supplementary figures.pdf]

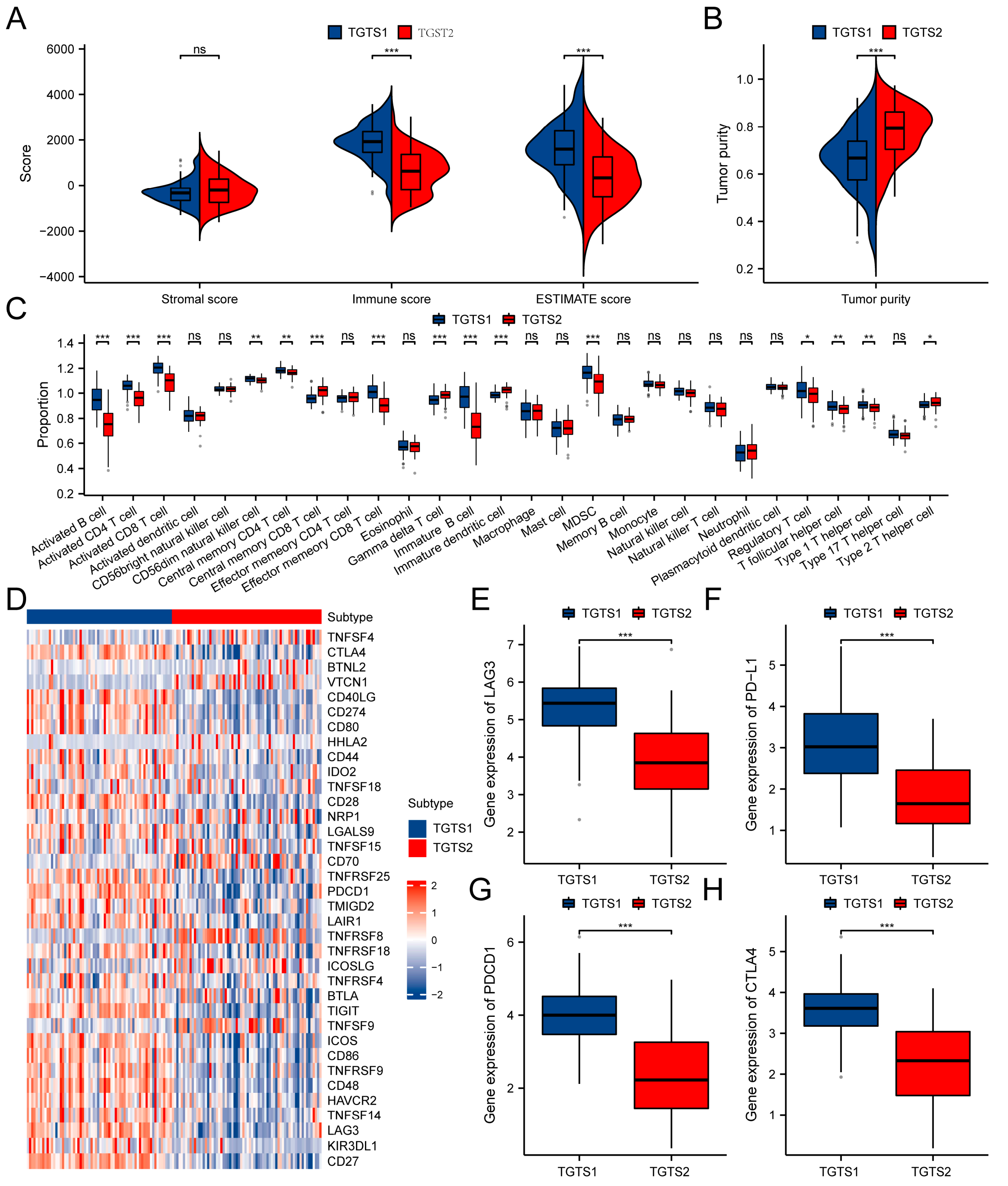

Supplementary Figure S1. Comparison of immune infiltration characteristics between subtypes. (A-B) The differences in the immune score (A), stromal score (A), ESTIMATE score (A), and tumor purity (B) among subtypes via the ESTIMATE algorithm. (C) Boxplot showed the difference in tumor-infiltrating immune cells among subtypes via the ssGSEA algorithm. (D) Heatmap of immune checkpoint genes among subtypes. (E-H) Boxplots showed the difference in gene expression of LAG3 (E), PD-L1 (F), PDCD1 (G), and CTLA4 (H). ns: $p \geq 0.05$; *: $p < 0.05$; **: $p < 0.01$; ***: $p < 0.001$.

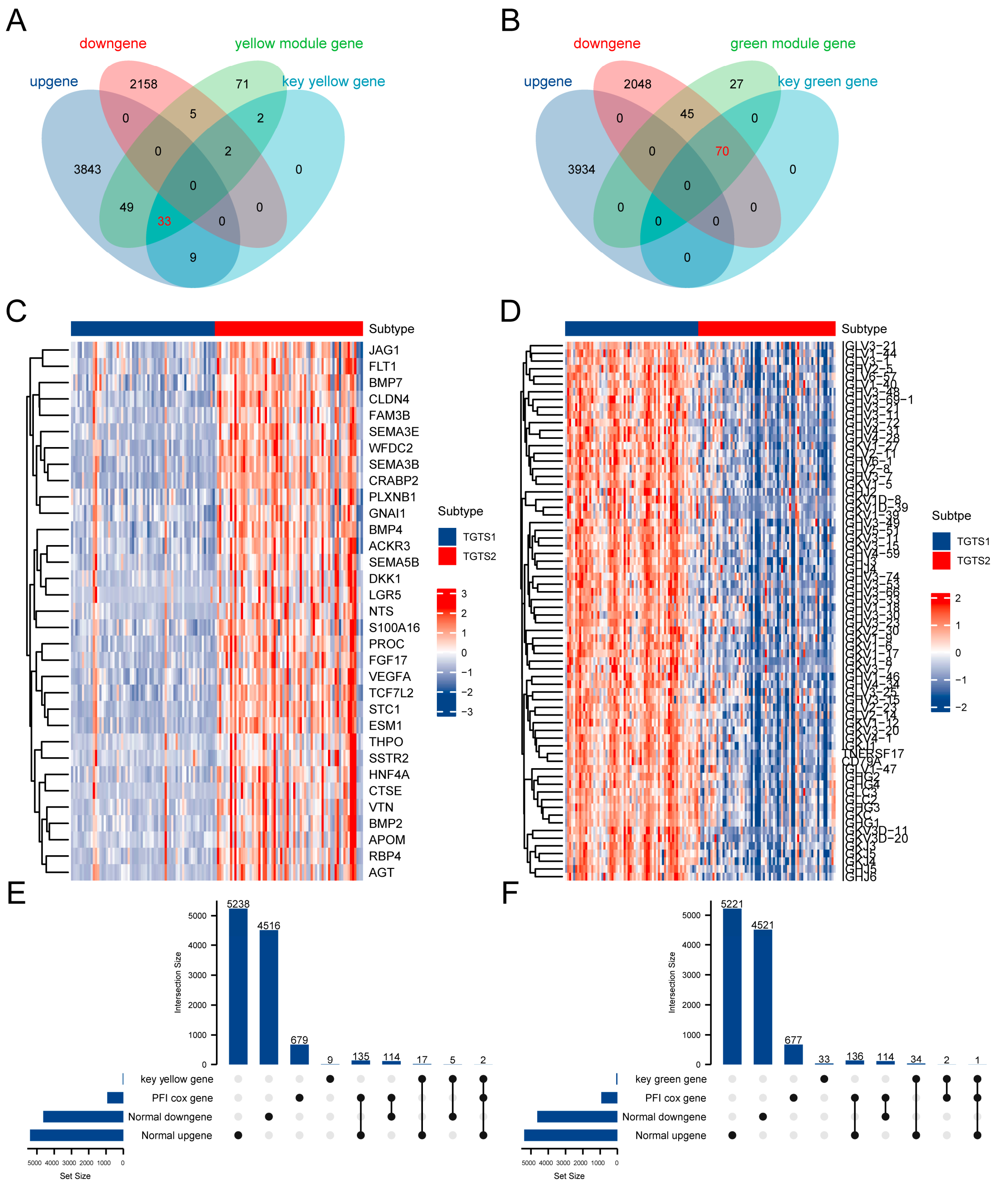

Supplementary Figure S2. Identification of potential diagnostic biomarkers. (A, B) Venn plots show the 33 up-regulated hub genes (A) and 70 down-regulated hub genes (B) between TGTS1 and TGTS2 based on the DEGs among subtypes, key module genes, and the key genes in the key modules. (C, D) Heatmaps of the 33 up-regulated hub genes (C) and 70 down-regulated hub genes (D) in the subtypes. (E, F) Upset plots of overlapping genes based on above hub genes, DEGs compared to normal samples, and PFI-related genes.

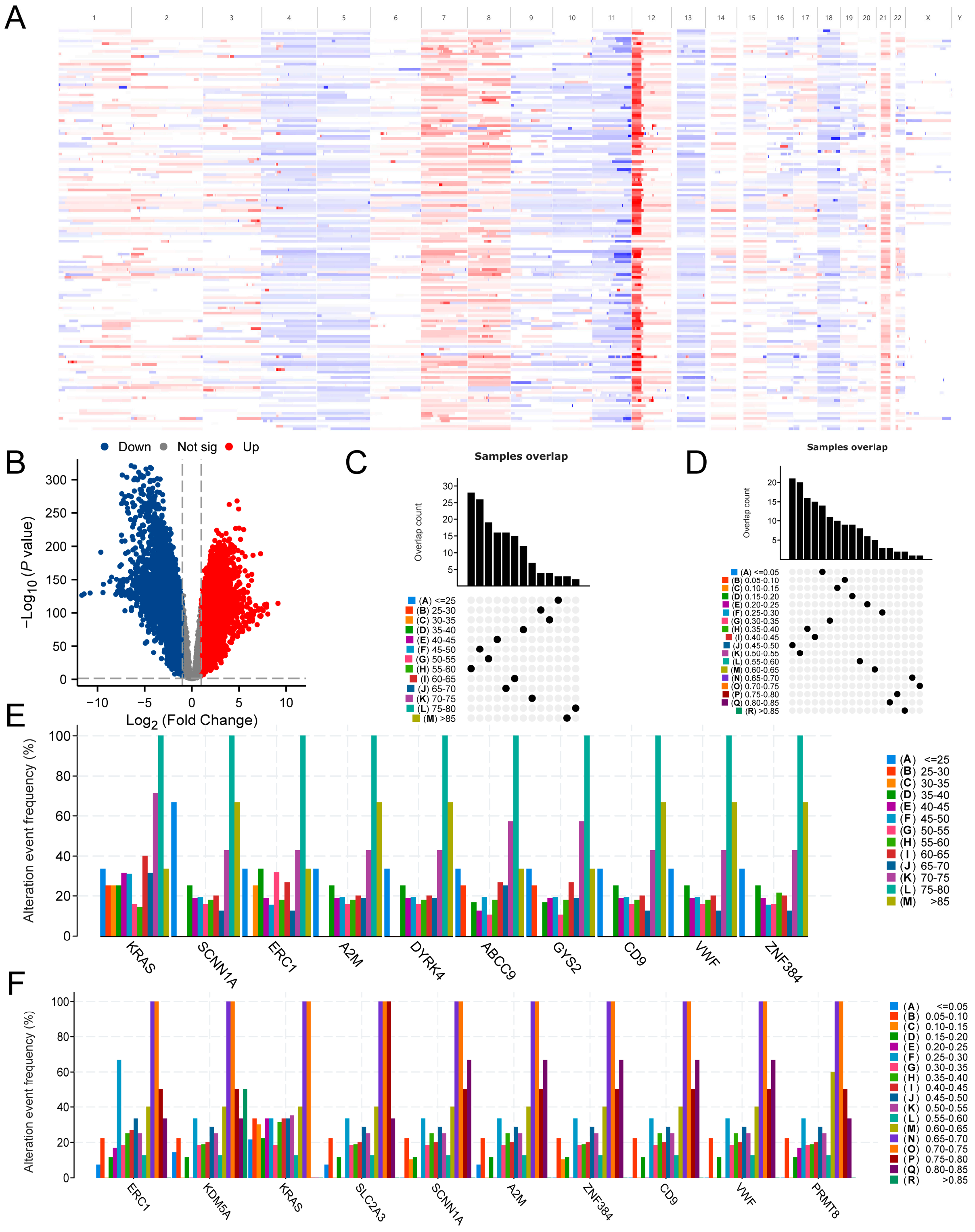

Supplementary Figure S3. Identification of candidate tumor therapeutic targets of TC. (A) Chromosomal distribution of aberrant copy number genes in TC. (B) Volcano plot of differentially expressed genes through R "limma" package. (C, D) Samples overlapping in the altered genome fraction group (C) and mutation count group (D). (E, F) Genes with the highest frequency in the altered genome fraction group (E) and mutation count group (F).

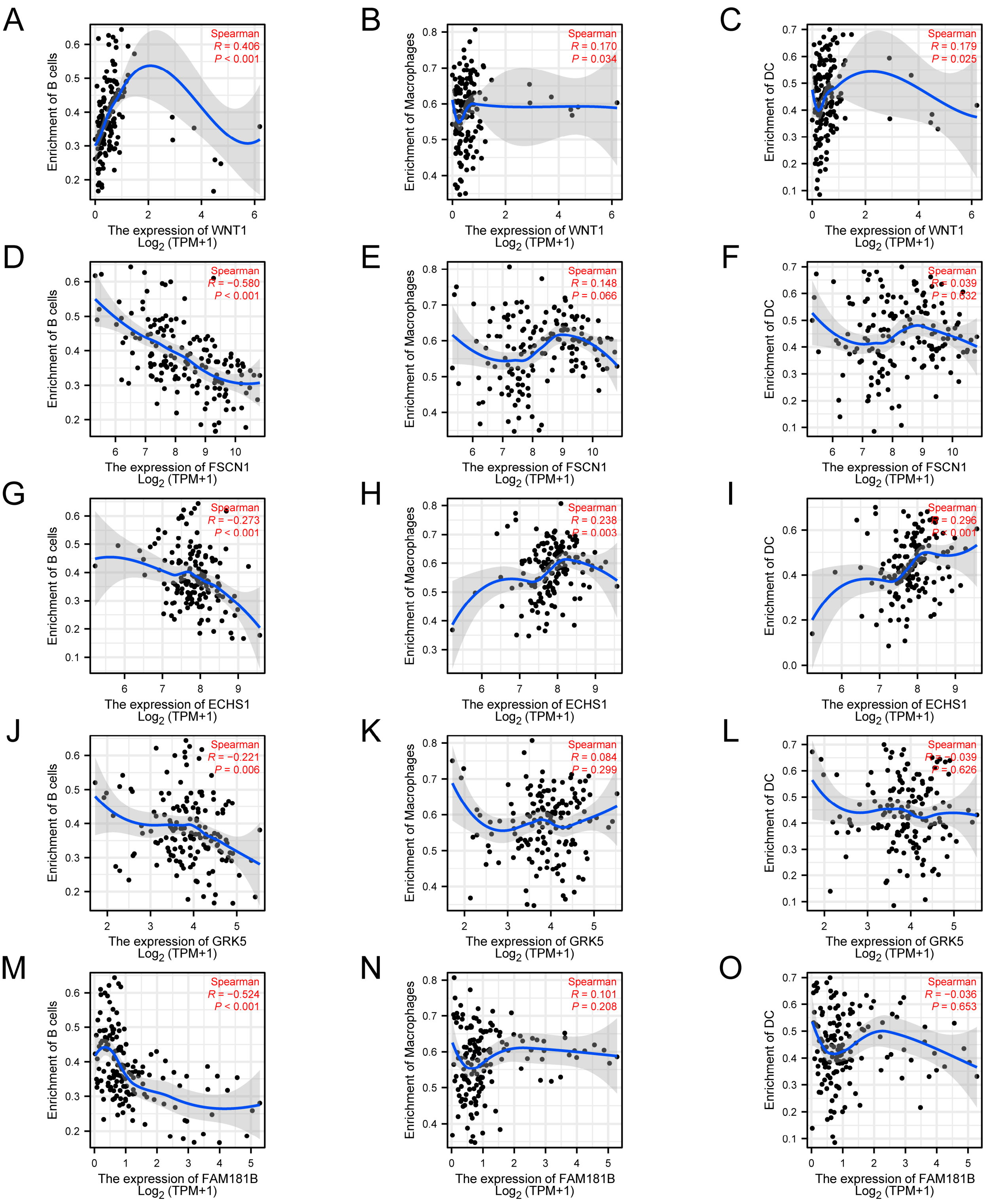

Supplementary Figure S4. Correlation between candidate tumor therapeutic targets and antigen-presenting cells. (A-O) The correlation between antigen-presenting cells (APCs) and WNT11 (A-C), FSCN1 (D-F), ECHS1 (G-I), GRK5 (J-L), and FAM181B (M-O) expression was calculated through Spearman's analysis.
